# Comparison of the prognosis of four different treatment strategies for acute left malignant colonic obstruction: a systematic review and network meta-analysis

**DOI:** 10.1186/s13017-021-00355-2

**Published:** 2021-03-18

**Authors:** Ling Tan, Zi-lin Liu, Meng-ni Ran, Ling-han Tang, Yan-jun Pu, Yi-lei Liu, Zhou Ma, Zhou He, Jiang-wei Xiao

**Affiliations:** 1 Department of Gastrointestinal Surgery, Clinical Medical College and The First Affiliated Hospital of Chengdu Medical College, Chengdu, 610500 Sichuan Province China; 2 State Key Laboratory of Biotherapy West China Hospital, West China Medical School, Sichuan University, Chengdu, 610500 Sichuan Province China

**Keywords:** Colonic stenting, Transanal colorectal tube, Decompressing stoma, Bridge to surgery, Emergency resection, Acute left malignant colonic obstruction, Network meta-analysis

## Abstract

**Background:**

There is controversy regarding the efficacy of different treatment strategies for acute left malignant colonic obstruction. This study investigated the 5-year overall survival (OS) and disease-free survival (DFS) of several treatment strategies for acute left malignant colonic obstruction.

**Methods:**

We searched for articles published in PubMed, Embase (Ovid), MEDLINE (Ovid), Web of Science, and Cochrane Library between January 1, 2000, and July 1, 2020. We screened out the literature comparing different treatment strategies. Evaluate the primary and secondary outcomes of different treatment strategies. The network meta-analysis summarizes the hazard ratio, odds ratio, mean difference, and its 95% confidence interval.

**Results:**

The network meta-analysis involved 48 articles, including 8 (randomized controlled trials) RCTs and 40 non-RCTs. Primary outcomes: the 5-year overall survival (OS) and disease-free survival (DFS) of the CS-BTS strategy and the DS-BTS strategy were significantly better than those of the ES strategy, and the 5-year OS of the DS-BTS strategy was significantly better than that of CS-BTS. The long-term survival of TCT-BTS was not significantly different from those of CS-BTS and ES. Secondary outcomes: compared with emergency resection (ER) strategies, colonic stent-bridge to surgery (CS-BTS) and transanal colorectal tube-bridge to surgery (TCT-BTS) strategies can significantly increase the primary anastomosis rate, CS-BTS and decompressing stoma-bridge to surgery (DS-BTS) strategies can significantly reduce mortality, and CS-BTS strategies can significantly reduce the permanent stoma rate. The hospital stay of DS-BTS is significantly longer than that of other strategies. There was no significant difference in the anastomotic leakage levels of several treatment strategies.

**Conclusion:**

Comprehensive literature research, we find that CS-BTS and DS-BTS strategies can bring better 5-year OS and DFS than ER. DS-BTS strategies have a better 5-year OS than CS-BTS strategies. Without considering the hospital stays, DS-BTS strategy is the best choice.

**Supplementary Information:**

The online version contains supplementary material available at 10.1186/s13017-021-00355-2.

## Background

Colorectal cancer (CRC) is ranked third and second among global cancer morbidity and mortality, respectively. The incidence rate of CRC is third in men and fourth in women [1]. Approximately 30% of CRC patients have acute colonic obstruction, and the overall prognosis is poor [2]. Compared with elective surgery for CRC without left-sided malignant colonic obstruction, emergency resection (ER) with left-sided malignant colonic obstruction is associated with a higher risk of mortality and morbidity [3].

Twenty years ago, colonic stent (CS) implantation was first used to restore the lumen opening of patients with malignant obstruction of the left colon as a bridge to surgery (BTS) [4]. The current clinical treatments for patients with malignant obstruction of the left colon include CS-BTS, transanal colorectal tube (TCT)-BTS, decompressing stoma (DS)-BTS, and ER. Research on the treatment of left obstructive colorectal cancer is gradually increasing. A meta-analysis showed that CS-BTS improved short-term surgical outcomes compared with ER but had similar long-term tumor and survival outcomes [5]. Compared with TCT-BTS, CS-BTS in the treatment of acute left malignant intestinal obstruction had a higher decompression efficiency, safety, and technical success rate; had fewer complications; and could avoid the formation of stoma [6]; moreover, DS-BTS has more primary anastomoses than ER [7]. TCT-BTS can increase primary resection/anastomosis compared to ER, but the long-term outcomes are similar [8]. Although many RCTs and many standard paired meta-analyses have been published to date, upon comparing the available treatment strategies for left obstructive colorectal cancer, there is still controversy regarding the best treatment strategy.

An important disadvantage of these RCTs and standard pairwise meta-analyses on this topic is that they can only directly compare two treatments, not all available treatments at once. A network meta-analysis can simultaneously compare all treatment strategies available for left obstructive colorectal cancer. Another advantage of network meta-analysis is that it combines direct and indirect evidence from trials to facilitate indirect comparisons between multiple treatments that have not been directly studied before and comparative studies [9, 10]. The purpose of this study was to conduct a systematic review of the literature to determine the relevant comparative treatment strategies available for left obstructive colorectal cancer, collect all published relevant data, and conduct a network meta-analysis to compare the long-term survival and short-term effects of the different treatment strategies.

## Methods

### Search strategy and inclusion criteria

A systematic search was performed based on the following databases: PubMed, Embase (Ovid), MEDLINE (Ovid), Web of Science, and Cochrane Library from January 1, 2000, to July 1, 2020. We used ‘colorectal cancer’, ‘obstruction’, ‘colonic stent’, ‘transanal colorectal tube’,’ decompressing stoma’, ‘bridge to surgery’, ‘emergency surgery’, and corresponding free words to search the literature in the above databases, with the language restricted to English (The search strategy are in Supplementary Table 1). This network meta-analysis only considers report research in the form of articles, both RCT and non-RCT. Non-RCT studies must use intention-to-treat analysis. To be included in the analysis, the article must compare two or more histologically confirmed treatment strategies for acute left malignant colonic obstruction, and the article must report at least one outcome of interest. If the study is based on the same database or patient population and reports the same results of interest, then unless the analysis is mutually exclusive, the reported results are different, or the results are measured, only the latest publications are included in the analysis. In the literature quality assessment, RCT literature is assessed based on Cochrane tools, and non-RCT literature is assessed based on Newcastle-Ottawa quality assessment Scale (NOS).

### Outcomes of interest

 1. Primary outcomes: 5-year overall survival (OS) and disease-free survival (DFS).

2. Secondary outcomes: primary anastomosis, mortality, anastomotic leak, permanent colostomy, and hospital stays.

### Data extraction

First, all the identified titles and abstracts were examined by two independent reviewers (TL and LZL). Next, the same two reviewers independently examined the full texts of potentially relevant articles. In the event of disagreement, a third reviewer (RMN) was consulted, and the relevant articles were discussed until a consensus was reached. The following relevant information was extracted from all the included publications: treatment strategy, country, number of patients, age, tumor grade, surgery, and follow-up. For long-term survival outcomes, if available, the following data were extracted: hazard ratios (HRs), 95% CI and *P* values of OS and DFS. When the literature did not report HRs, only OS and DFS K-M curves, Engauge Digitizer (version 10.8) was used to determine the survival rates of the corresponding time points on the curve, followed by the HR calculation table [11]. All the data were independently extracted by two authors (TL and LZL) and compared for consistency.

### Statistical analysis

For each result of interest, we used STATA (version 15.3) to draw a network diagram of all treatments evaluated for that particular result. The network meta-analysis was performed using the Markov chain Monte Carlo method in WinBUGS 1.4. The results of the network meta-analysis involved the measurement of central tendency and post-standard deviation or confidence interval (CI). For binary results, the binomial model was used for analysis and the odds ratios (ORs) were calculated. For continuous results, the mean difference (MD) was calculated. For long-term results, the survival analysis model was used to calculate the HR. Modelling the treatment comparison between any two treatments (OR for binary results, MD for continuous results, and HR for long-term results) depends on the comparison between each individual treatment and an arbitrarily selected reference treatment. The reference treatment was chosen as the open method, and the likelihood of the treatment level (i.e., the treatment is rated as the best treatment, suboptimal treatment, suboptimal treatment, etc.) for each outcome of interest was calculated. The authors believe that a ranking probability of less than 90% is not high enough to be confidently reported as the correct ranking of surgical techniques of interest to this result [12].

We used residual deviation and deviation information criteria (DIC) to assess the heterogeneity between studies. We used three different models for each result: a fixed effects model, a random effects model, and a random effects inconsistent model. Model selection was based on model fitting. DIC provided a measure of model fit. If the DIC values between the fixed-effects model and the random-effects model were similar, a simpler model, the fixed-effects model, was used; if the fit of the random model represented by DIC was at least 3 lower than the fit of the fixed-effects model, the random-effects model was used [13, 14]. The data were evaluated for evidence of inconsistency between direct and indirect comparisons by examining the geometry [13, 14]. In addition, the deviation and DIC statistics of consistent and inconsistent models were compared. If the inconsistent model had a better model fit than the consistent model, the network meta-analysis should be interpreted with caution [13–15].

## Results

**Fig. 1:**
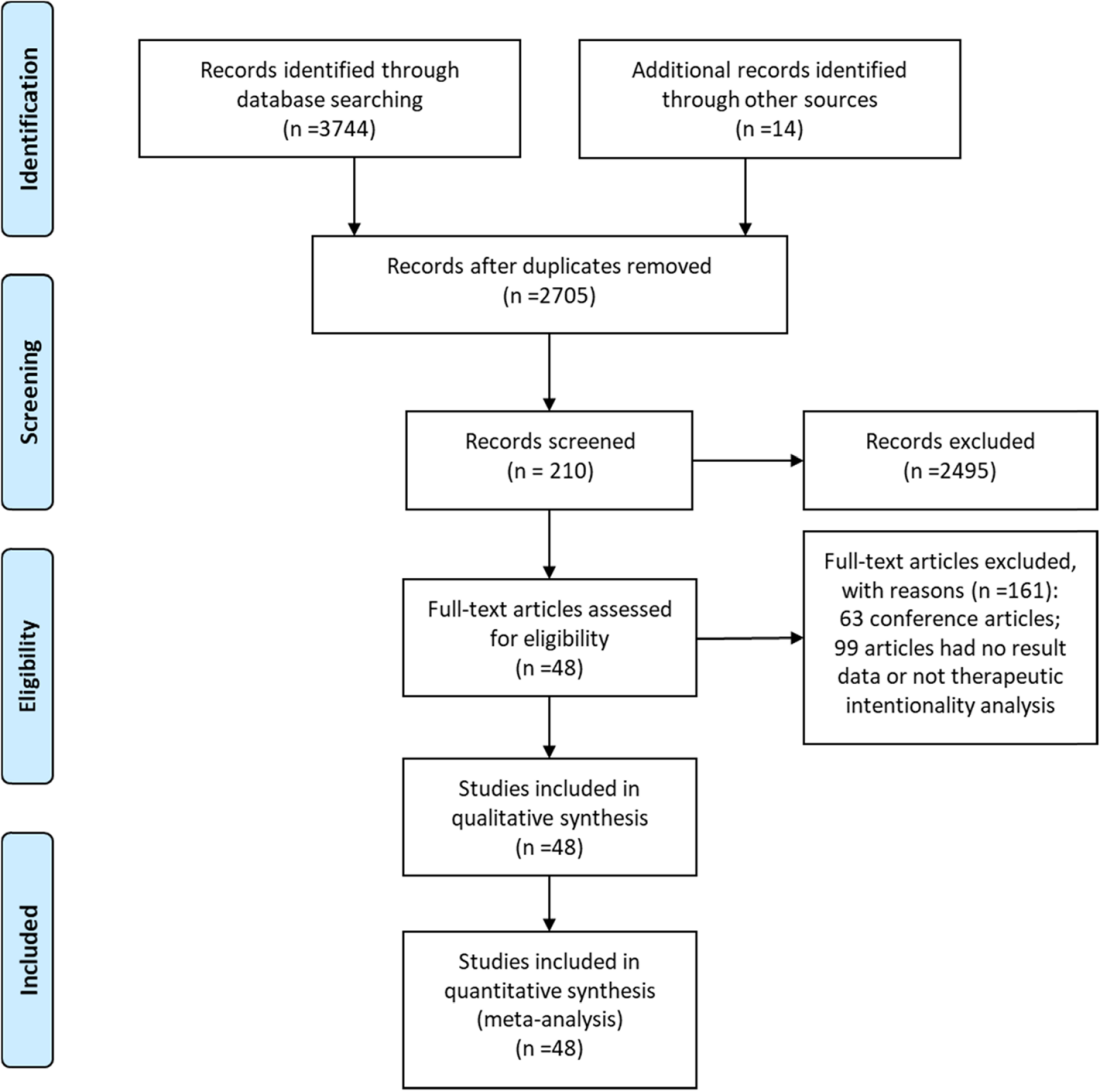
Flowchart of search strategy and study selection

Our computer-aided search yielded 2705 publications from PubMed, MEDLINE (Ovid), Embase (Ovid), Web of Science, and Cochrane Library after removing the duplicate literature. By screening the titles and reading abstracts, we excluded another 2495 obviously irrelevant documents. Further full-text screening of 210 publications was carried out, and 161 articles were excluded (Fig. 1). Ultimately, this network analysis contained 48 articles, including 7 RCT experiments (8 RCT literatures) [16–23] and 40 non-RCT experiments [8, 24–62]. The characteristics of the included studies (first author, journal, country, treatment strategy, basic characteristics of the study population, etc.) are summarized in Table 1. The risk of bias and literature quality assessment of each study included in the analysis are summarized in Supplementary Table 2. For RCT experiments, the risk of bias tool based on the Cochrane collaboration found that the quality of the included trials met the research standards. For non-RCT experiments, a NOS score of 7–9 indicates that the quality of the included trials meets the research standards.

**Table 1:** Characteristics of studies included in network meta-analysis of four different treatment strategies for the treatment of acute left malignant colonic obstruction

Author and year of publication	Journal	Treatment strategy	Countries	Sample size	Age	Stage	Operation method	Follow-up (month)	Study design
Okuda, 2019 [8]	Cancer Res Treat	TCT-BTS Vs ES	Japan	46	72 VS 70	Stage II-III	Laparoscopic and laparotomy	60	no-RCT
Jiang 2008 [62]	Dis Colon Rectum	DS-BTS Vs ES	China	143	68.2 VS 67.4	Stage I-III	–	120	no-RCT
Amelung, 2016 [51]	Surg Endosc	CS-BTS Vs DS-BTS	Dutch	88	71.8 VS 66.6	Stage I-IV	Laparoscopic and laparotomy	46	no-RCT
Mege, 2019 [52]	Ann Surg Oncol	CS-BTS Vs DS-BTS	France	518	72 VS 71	Stage I-IV	–	140	no-RCT
Veld, 2020 [53]	JAMA Surgery	CS-BTS Vs DS-BTS	Dutch	242	70.1 VS 69.8	uncertain	Laparoscopic and laparotomy	32	no-RCT
Kagami, 2018 [58]	World J Surg Oncol	CS-BTS Vs TCT-BTS	Japan	59	70 VS 68	Stage II-IV	Laparoscopic and laparotomy	–	no-RCT
Sato 2019 [59]	Ann Gastroenterol Surg	CS-BTS Vs TCT-BTS	Japan	76	70.8 VS 76.0	Stage II-III	Laparoscopic and laparotomy	30	no-RCT
Yang, 2019, [60]	Oncology Letters	CS-BTS Vs TCT-BTS	China	89	50.64 VS 52.04	Stage I-IV	Laparoscopic and laparotomy	12	no-RCT
Hosono 2019 [57]	Asian J Endosc Surg	CS-BTS Vs TCT-BTS	Japan	42	74 VS 74	Stage II-IV	Laparoscopic and laparotomy	21	no-RCT
Kawachi, 2018 [61]	ASIAN J SURG	CS-BTS Vs TCT-BTS Vs ES	Japan	56	69.4 VS 74.1 VS 68.9	Stage II-IV	-	–	no-RCT
Amelung, 2016 [54]	Ann Surg Oncol	CS-BTS Vs DS-BTS Vs ES	Dutch	1860	69.9 VS 64.9 VS 71.4	uncertain	Laparoscopic and laparotomy	–	no-RCT
Oistamo, 2016 [55]	World J Surg Ocol	CS-BTS Vs DS-BTS Vs ES	Sweden	100	71 VS 67 VS 74	Stage II-IV	-	–	no-RCT
Tanis, 2015 [56]	Digestive Surgery	CS-BTS Vs DS-BTS Vs ES	Dutch	1816	71 VS 68 VS 70	Stage I-IV	Laparoscopic and laparotomy	–	no-RCT
Amelung, 2017 [24]	Surg Endosc	CS-BTS Vs ES	Dutch	110	70VS70.4	Stage II-IV	Laparoscopic and laparotomy	44	no-RCT
Arezzo, 2017 [16]	Surg Endosc	CS-BTS Vs ES	Italy	115	72 VS 71	uncertain	Laparoscopic and laparotomy	36	RCT
Chen, 2019 [25]	World JGastroenterol	CS-BTS Vs ES	China	128	63.21 VS 61.58	Stage I-IV	–	-	no-RCT
Choi, 2014 [26]	Surg Endosc	CS-BTS Vs ES	Korea	240	65.2 VS 64.8	Stage II-IV	–	41.4 VS 45	no-RCT
Consolo, 2017 [27]	Turk J Gastroenterol	CS-BTS Vs ES	Italy	125	74.2 VS 70	Stage I-IV	–	–	no-RCT
Erichsen, 2015 [29]	Endoscopy	CS-BTS Vs ES	Denmark	3914	–	–	–	24	no-RCT
Flor-Lorente, 2017 [30]	Cirugia Espanola	CS-BTS Vs ES	Spain	82	72 VS 70	Stage I-IV	Laparoscopic and laparotomy	58	no-RCT
Ghazal, 2013 [18]	J Gastrointest Surg	CS-BTS Vs ES	Egypt	60	52VS51	Stage I-III	Laparotomy	18	RCT
Gorissen, J.2013 [31]	Br J Surg	CS-BTS Vs ES	England	105	70.6VS72.0	Stage I-IV	Laparoscopic and laparotomy	32VS33	no-RCT
Han, 2020 [33]	PAK J MED SCI	CS-BTS Vs ES	China	302	60.25VS 61.03	Stage II-IV	-	45.82VS44,92	no-RCT
Ho, 2017 [34]	Surgical Endoscopy	CS-BTS Vs ES	China	102	70.2 VS 70.9	Stage I-IV	Laparoscopic and laparotomy	21 VS 25.5	no-RCT
Ho, 2012 [19]	Int J Colorectal Dis	CS-BTS Vs ES	Singapore	39	68 VS 65	Stage II-IV	Laparoscopic and laparotomy	–	RCT
Kavanagh, 2013 [35]	Dis Colon Rectum	CS-BTS Vs ES	Ireland	49	69.9 VS 69.7	Stage I-III	Laparoscopic and laparotomy	27.4 VS 26	no-RCT
Kim, 2016 [36]	ANZ J Surg	CS-BTS Vs ES	Korea	168	64.6 VS 64.5	uncertain	Laparoscopic and laparotomy	45 VS 49.5	no-RCT
Kim, 2015 [37]	Surg Endosc	CS-BTS Vs ES	Korea	56	64.6 VS 70.7	Stage II-IV	Laparoscopic and laparotomy	30 VS 26	no-RCT
Kwak, 2016 [38]	Dis Colon Rectum	CS-BTS Vs ES	Korea	84	62 VS 60	Stage I-IV	Laparoscopic and laparotomy	44	no-RCT
Lee, 2013 [39]	Int J Surg	CS-BTS Vs ES	Korea	77	63.6 VS 56.6	Stage I-IV	Laparoscopic and laparotomy	38.7	no-RCT
Lim, 2017 [40]	Ann Surg Oncol	CS-BTS Vs ES	Singapore	102	65 VS 66	Stage I-III	Laparoscopic and laparotomy	48	no-RCT
Lovero, 2020 [41]	Eur J Clin Invest	CS-BTS Vs ES	Italy	45	64.7 VS 71.2	Stage I-IV	Laparoscopic and laparotomy	15	no-RCT
Morita, 2019 [42]	Surg Today	CS-BTS Vs ES	Japan	201	74 VS 70	Stage I-IV	Laparoscopic and laparotomy	–	no-RCT
Park, 2018 [44]	Int J Colorectal Dis	CS-BTS Vs ES	Korea	111	64 VS 69	Stage I-IV	Laparoscopic and laparotomy	58.2 VS 50.4	no-RCT
Park, 2016 [45]	Ann Surg Oncol	CS-BTS Vs ES	Korea	102	68.6 VS 63.1	Stage I-III	Laparoscopic and laparotomy	35.7 VS 46.6	no-RCT
Pirlet, 2011 [20]	Surg Eedosc	CS-BTS Vs ES	France	60	70.4 VS 74.7	uncertain	Laparotomy	–	RCT
Rodrigues-Pinto, 2019 [46]	Dig Liver Dis	CS-BTS Vs ES	Italy	94	67 VS 75	Stage I-IV	Laparoscopic and laparotomy	24 VS 30	no-RCT
Sabbagh, 2013 [47]	Ann Surg	CS-BTS Vs ES	France	87	69.73 VS 74.89	Stage I-IV	Laparoscopic and laparotomy	28 VS 32	no-RCT
Sloothaak, 2014 [21]	Br J Surg	CS-BTS Vs ES	Dutch	58	70 VS 67	Stage I-IV	–	45 VS 41	RCT
van den Berg, 2014 [48]	Br J Surg	CS-BTS Vs ES	Dutch	110	71 VS 72	Stage I-IV	Laparoscopic and laparotomy	–	no-RCT
Yan, 2017 [49]	J Laparoendosc Adv Surg Tech A	CS-BTS Vs ES	China	60	60.44 VS 59.36	Stage II-IV	–	–	no-RCT
Yang, 2019 [50]	Ann Surg Oncol	CS-BTS Vs ES	Korea	253	65.2 VS 63.9	Stage I-III	Laparoscopic and laparotomy	60.4 VS 53.4	no-RCT
van Hooft 2011 [23]	The lancet	CS-BTS Vs ES	Dutch	98	70.4 VS 71.4	uncertain	-	39 VS 44	RCT
Cheung, 2009/2012 [17, 22]	Arch Surg/Asian J Endosc Surg	CS-BTS Vs ES	China	48	64.5 VS 68.5	Stage I-IV	Laparoscopic and laparotomy	65 VS 32	RCT
Guo 2011 [32]	Dig Dis Sci	CS-BTS Vs ES	China	92	77 VS 76	uncertain	-	–	no-RCT
Ng 2006 [43]	J Gastrointest Surg	CS-BTS Vs ES	China	60	74 VS 73.5	Stage II-IV	Laparoscopic and laparotomy	–	no-RCT
Dastur 2008 [28]	Tech Coloproctol	CS-BTS Vs ES	England	42	75 VS 68	uncertain		21 VS 30	no-RCT

### Overall analysis

**Fig. 2:**
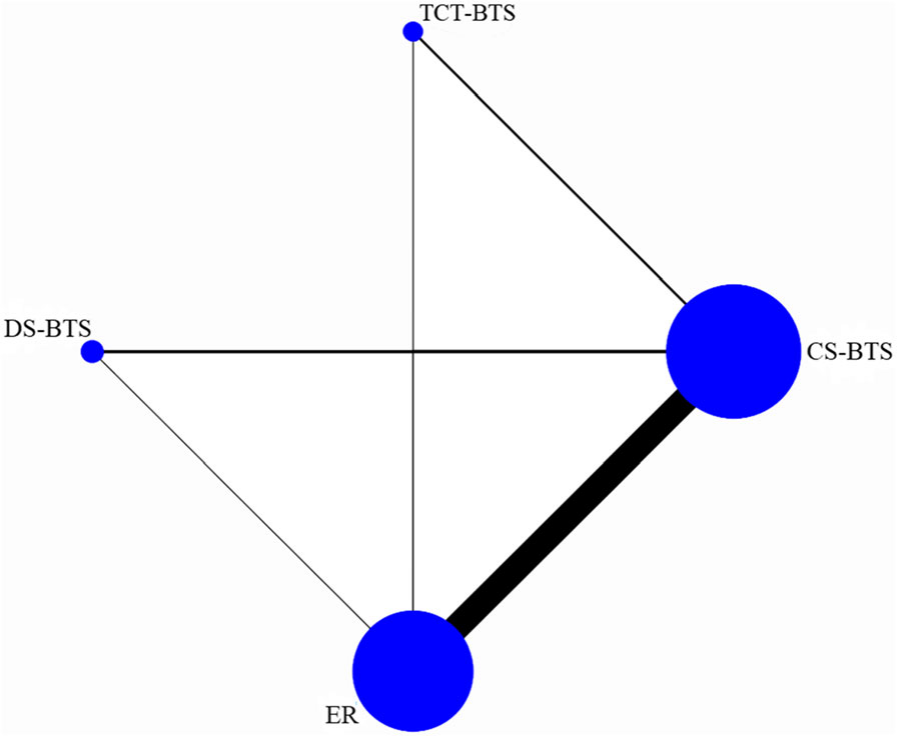
The network diagram of primary anastomosis

There were 35 [16–50] studies comparing CS-BTS and ER treatment strategies, 6 [51–56] studies comparing CS-BTS and DS-BTS treatment strategies, 5 [57–61] studies comparing CS-BTS and TCT-BTS treatment strategies, 4 [54–56, 62] studies comparing DS-BTS and ER treatment strategies, and 2 [8, 61] studies comparing TCT-BTS and ER treatment strategies. A total of 12,514 patients received 4 different treatment strategies: 3058 CS-BTS, 153 TCT-BTS, 775 DS-BTS, and 8528 ER. Figure 2 shows the network diagram of primary anastomosis. Similar network diagrams were constructed for all the results of interest. For all the results of interest, there was no evidence of inconsistency between the trials in the network because the DIC differences between the consensus model and the inconsistency model were not significant. The treatment strategies ranked from best to worst (1st to 4th) for the outcome of interest are summarized in Table 2. Among the four treatment strategies, the treatment strategy with the least primary anastomosis may be ER (95% probability ER ranks 4th), while the best treatment strategy for 5-year OS was DS-BTS (95% probability DS-BTS ranks 1st). The treatment strategy with the longest hospital stay was DS-BTS, and the treatment strategy with the shortest hospital stay was TCT-BTS (100% probability DS-BTS ranks 1st, and 100% probability TCT-BTS ranks 4th).

**Table 2:** Probability of Ranking From Best to Worst (1st–4th) for the Outcomes of Interest

Outcomes	Ranks			
	1st	2nd	3rd	4th
Primary anastomosis	TCT-BTS *P*=0.46	CS-BTS *P*=0.48	DS-BTS *P*=0.63	**ER** ***P*=0.95**
Mortality	ER *P*=0.68	TCT-BTS *P*=0.38	CS-BTS *P*=0.65	DS-BTS *P*=0.76
Anastomotic leak	TCT-BTS *P*=0.59	ER *P*=0.53	CS-BTS *P*=0.54	DS-BTS *P*=0.62
Permanent colostomy	ER *P*=0.77	TCT-BTS *P*=0.52	CS-BTS *P*=0.5	DS-BTS *P*=0.48
Hospital stays	**DS-BTS** ***P*=1**	**ER** ***P*=0.95**	**CS-BTS** ***P*=0.95**	**TCT-BTS** ***P*=1**
Five-year DFS	DS-BTS *P*=0.68	CS-BTS *P*=0.673	ER *P*=0.56	TCT-BTS *P*=0.57
Five-year OS	**DS-BTS** ***P*=0.95**	CS-BTS *P*=0.87	ER *P*=0.72	TCT-BTS *P*=0.72

Ranking with more than 90% probability is highlighted in bold. A probability of ranking below 90% was not considered by the authors to be high enough to be confidently reported as the correct ranking position of a treatment strategy for that outcome of interest

### Primary outcomes

Table 3 shows a pairwise comparison of the long-term results of several different treatment strategies (CS-BTS, TCT-BTS, DS-BTS and ER). For the 5-year overall survival rate, the evidence indicates that CS-BTS and DS-BTS are significantly better than ER (ER Vs CS-BTS, DS-BTS, HR are 1.14 (1.04–1.26), 1.29 (1.13–1.48), respectively), and DS-BTS is significantly better than CS-BTS (DS-BTS Vs CS-BTS HR are 0.88 (0.80–0.98)). For the 5-year DFS, the evidence indicates that CS-BTS and DS-BTS are significantly better than ER (ER Vs CS-BTS, DS-BTS, HR are 1.12 (1.06–1.35), 1.23 (1.06–1.44), respectively). Compared with the other three treatment strategies, the TCT-BTS strategy had no significant differences in five-year OS and DFS.

**Table 3:** Pairwise comparisons for 5-year survival outcomes

DFS	CS-BTS	TCT-BTS	DS-BTS	ER
		1.23 (0.88–1.72)	0.97 (0.88–1.07)	1.12 (1.06–1.35)
	TCT-BTS	–	0.79 (0.56–1.12)	0.97 (0.70–1.36)
	DS-BTS	–	–	**1.23 (1.06**–**1.44)**
OS	CS-BTS	1.29 (0.85–1.97)	**0.88 (0.80-0.98)**	**1.14 (1.04**–**1.26)**
	TCT-BTS	–	0.68 (0.45–1.05)	0.89 (0.59–1.34)
	DS-BTS	–	–	**1.29 (1.13**–**1.48)**

Hazard ratio horizontal treatment over vertical treatment (95% credible intervals CI)

### Secondary outcomes

Table 4 shows a pairwise comparison of short-term postoperative outcomes between different treatment strategies. Paired comparison results showed that there was no significant difference in postoperative anastomotic leakage with different treatment strategies. Compared with ER, CS-BTS and TCT-BTS strategies can significantly increase the one-stage anastomosis rate; compared with ER, CS-BTS, and DS-BTS strategies can significantly reduce mortality; compared with ER, CS-BTS can significantly reduce the rate of permanent stoma. In addition, the longest hospital stay was with DS-BTS, and the shortest was with TCT-BTS.

**Table 4:** Pairwise comparisons for short-term postoperative outcomes

		TCT-BTS	DS-BTS	ER
Primary anastomosis*	CS-BTS	0.98 (0.26–3.71)	0.61 (0.22–1.68)	**0.23 (0.13**–**0.38)**
	TCT-BTS	–	0.63 (0.12-3.05)	**0.23 (0.06**–**0.84)**
	DS-BTS	–	–	0.37 (0.13–1.06)
Mortality*	CS-BTS	1.48 (0.29–6.29)	0.71 (0.35-1.23)	**2.13 (1.59**–**3.22)**
	TCT-BTS	–	0.48 (0.10-2.61)	1.45 (0.35–8.01)
	DS-BTS	–	–	**3.03 (1.75**–**6.67)**
Anastomotic leak*	CS-BTS	1.69 (0.35–7.88)	0.75 (0.22–2.21)	1.33 (0.84–2.21)
	TCT-BTS	–	0.45 (0.07–3.11)	0.79 (0.17–3.89)
	DS-BTS	–	–	1.77 (0.61–6.11)
Permanent colostomy*	CS-BTS	1.89 (0.50–7.14)	0.98 (0.27–3.51)	**3.28 (1.75**–**6.41)**
	TCT-BTS	–	0.52 (0.08–3.34)	1.75 (0.45–6.77)
	DS-BTS	–	–	3.35 (0.88–14.07)
Hospital stays†	CS-BTS	**−15.35 (−25.43**–**5.13)**	**13.76 (9.13**–**18.03)**	2.10 (**−**0.44–5.27)
	TCT-BTS	–	**29.00 (18.02**–**39.73)**	**17.46 (6.24**–**27.77)**
	DS-BTS	–	–	**−11.58 (−15.60**–**6.77)**

Statistically significant outcomes in bold: OR was significant if the 95% CI did not include the value 1, MD was significant if the 95% CI did not include the value 0

^*^Odds ratio of horizontal treatment over vertical treatment

^†^Mean difference of horizontal treatment minus vertical treatment, (95% credible intervals CI)

## Discussion

This is the first network meta-analysis that can simultaneously compare several different treatment strategies for left-sided malignant colonic obstruction. The European Society of Gastrointestinal Endoscopy 2020 Guidelines recommend colon stents as a bridge to elective surgery in acute malignant obstruction of the left colon; at the same time, when the patient is not suitable for colonic stent placement, or when there is no professional for stent placement, decompression stoma is an effective choice as a bridge for selective surgery [63]. This network meta-analysis showed that patients with CS-BTS and DS-BTS strategies had a better prognosis than patients with ER strategies, while patients with DS-BTS strategies had better OS than patients with CS-BTS strategies. The previous standard paired meta-analysis and RCT comparison between CS-BTS and ER in the treatment of acute left colonic malignant obstruction proved that although CS-BTS increased the hospital stay, and it also increased the primary anastomosis rate. At the same time, postoperative complications, anastomotic leak, short-term mortality, wound infection, initial stoma, and permanent stoma were significantly reduced [16, 17, 19, 20, 64–69]. Similarly, our research proves that the CS-BTS strategy can increase primary anastomosis and reduce permanent stoma and short-term mortality compared with the ER strategy. Previous paired meta-analyses and RCT comparisons of CS-BTS and ER in the treatment of acute left colonic malignant obstruction proved that the long-term results are similar [5, 16, 21, 22, 70–74], but our study proved that the long-term survival of CS-BTS is better than that of ER. The reasons for our analysis are as follows: ER often belongs to the state of incomplete surgical preparation, the general nutritional and immune status of patients is worse, and it is more likely to lead to tumor recurrence; longer recovery time after ER may lead to delayed chemotherapy; ER pays more attention to speed while neglecting lymph node dissection. Under these comprehensive factors, the long-term survival rate of ER is even worse. At same time, it may also be because we included more studies and the analysis method of the included studies adopted an intention-to-treat analysis.

Early research shows that, compared with the ER strategy, the DS-BTS strategy is a safe and effective treatment for acute left obstructive colon cancer [7]. Although the DS-BTS strategy had the same early mortality, complications, and anastomotic leakage as the ER strategy, the DS-BTS strategy increased the length of hospital stay and resulted in a significant increase in primary anastomosis and a significant decrease in permanent stoma [7, 54, 55, 62]. This network meta-analysis found that the anastomotic leakage and length of hospital stay were similar in the previous study, but our study showed that the early mortality rate of the DS-BTS strategy was significantly lower than that of the ER strategy. The difference in this strategy may be that DS-BTS is divided into two operations, the first operation is relatively small, and the surgical trauma is relatively small, so the early mortality rate is relatively low. The DS-BTS strategy requires two operations, which increases the patient’s hospital stay and costs [75]. Our study proved that the 5-year OS and DFS of the DS-BTS strategy are significantly better than those of the ER strategy. The DS-BTS strategy increases the patient’s hospital stay and costs but returns good long-term survival. Previous studies have proved that compared with the ER strategy, the DS-BTS strategy can significantly increase the production of lymph nodes [55], which is an important prognostic factor for colorectal malignancies. Perhaps because of this increased lymph node production, the obstructive colorectal cancer 5-year OS is significantly increased. This may be one of the important reasons why DS-BTS strategy leads to better 5-year OS and DFS than ER strategy.

Previous meta-analyses and RCTs showed that the CS-BTS strategy has fewer complications, a lower stoma rate, a higher primary anastomosis rate, and higher technical and clinical success rates than the TCT-BTS strategy in the treatment of acute left malignant intestinal obstruction [6, 76, 77]. Perhaps because the guide wire of the stent is small, it is easy to pass through the narrow part of the region. At the same time, when the stent is placed, the guide wire is more likely to make the stent reach the front end of the tumor [6], resulting in higher technical and clinical success rates of the CS-BTS strategy than the TCT-BTS strategy. Compared with the CS decompression strategy, the TCT strategy has an equivalent decompression effect [78]. However, patients with the TCT-BTS strategy require long-term retention of the anal decompression tube in the anus, which is associated with a great psychological burden and a bad mental state, which may be the reason for the worse prognosis compared to CS-BTS. Compared with the ER strategy, the TCT-BTS strategy has similar permanent stoma, short-term mortality, and long-term survival rates, but it increases the primary anastomosis rate [8, 61]. This network meta-analysis showed that there were no significant differences between the CS-BTS strategy and the TCT-BTS strategy in terms of primary anastomosis, mortality, anastomotic leakage, permanent stoma, and long-term survival. At the same time, the TCT-BTS and ER strategies have similar permanent stoma rates, short-term mortality, and long-term survival, and the increased primary anastomosis rates are similar to previous studies.

Compared with elective surgery for CRC without left malignant colon obstruction, emergency surgery with left malignant colon obstruction usually requires multiple operations, prolongs the hospital stay, and is associated with higher mortality and morbidity [3]. Current research shows that for curable acute left-sided malignant colonic obstruction patients, CS and DS are both effective decompression methods [51], but there is still controversy regarding the prognosis of tumors [52–54]. At the same time, the DS-BTS strategy requires more temporary colostomies and incisional hernias [51]. The DS-BTS strategy requires more surgical procedures to reinstate the stoma and repair the incision hernia. This network meta-analysis found that all short-term results of CS-BTS and DS-BTS were similar, except that the hospital stay in DS-BTS was longer. However, the long-term result is that the 5-year OS of the DS-BTS strategy is significantly better than that for the CS-BTS strategy. DS-BTS offers a better long-term survival than CS-BTS, which can be accompanied by some serious complications, including stent re-blocking, displacement, and intestinal perforation [79], and ER might be necessary, which could cause a poor prognosis. The abovementioned complications after DS-BTS decompression are relatively rare. It is worthwhile to implement a DS-BTS strategy for a better 5-year survival, notwithstanding a longer hospital stay and reoperation rate due to DS-BTS.

This network meta-analysis yielded short-term results similar to those of previous studies. In addition, we also obtained the long-term survival results of several treatment strategies. CS-BTS and DS-BTS significantly increased the 5-year OS compared with ER. Early large-scale studies and RCTs have shown that for acute left colonic obstructive malignant tumors, surgery after the remission of intestinal obstruction can significantly improve the short-term outcome (mortality [19, 54], primary anastomosis [8], stoma [16, 17, 20, 61]). For example, after the intestinal obstruction is relieved, the decrease of the stomata rate is related to the relief of oedema in the intestinal tract [80]. It is possible that relief of edema in the intestinal tract can increase primary anastomoses, decrease anastomotic leakage, and decrease mortality. Finally, it seems that in the treatment of acute left obstructive colonic malignancies, preoperative removal of the obstruction can improve the patient’s 5-year OS and DFS (whether it is CS-BTS or DS-BTS), possibly because the removal of intestinal obstruction can improve the patient’s nutritional status, enhance immunity, provide the opportunity to prepare the bowel, reduce the state of inflammatory stress, and increase the tumor R0 resection rate and more thorough lymph node dissection.

This research involves several limitations that must be considered. Since there are only 8 RCTs in the included 48 articles, and the RCT studies compared CS-BTS and ES, other treatment strategy studies are non-RCTs, which may cause some deviations in the results. The inclusion criteria limit the need for intentional analysis; this deviation should be minimized as much as possible.

## Conclusion

In comprehensive literature research, we find that CS-BTS and DS-BTS strategies can bring better 5-year OS and DFS than ER. DS-BTS strategies have a better 5-year OS than CS-BTS strategies. Without considering the hospital stays, DS-BTS strategy is the best choice.

## Supplementary Information

**Additional file 1: Table 1**. Literature search strategy. **Table 2**. Assessment of Methodological Quality of Studies Included

## Data Availability

The datasets used and analyzed during the current study are available from the corresponding author on reasonable request.

## Abbreviations

CRC: Colorectal cancer
RCT: Randomized controlled trial
ER: Emergency resection
CS: Colonic stent
BTS: Bridge to surgery
TCT: Transanal colorectal tube
DS: Decompressing stoma
CS-BTS: Colonic stent-bridge to surgery
TCT-BTS: Transanal colorectal tube-bridge to surgery
DS-BTS: Decompressing stoma-bridge to surgery
NOS: Newcastle-Ottawa quality assessment Scale
DFS: Disease-free survival
OS: Overall survival
HR: Hazard ratio
CI: Confidence interval
MD: Mean difference
OR: Odds ratio
DIC: Deviation information criteria
