# Supplementary material for: Comparison of the prognosis of four different treatment strategies for acute left malignant colonic obstruction: a systematic review and network meta-analysis
Source: World J Emerg Surg. 2021 Mar 18;16:11. doi: 10.1186/s13017-021-00355-2 (PMC7977175; doi:10.1186/s13017-021-00355-2)
Supplement: Supplementary file 1 — Additional file 1: Table 1. Literature search strategy. Table 2. Assessment of Methodological Quality of Studies Included [file 13017_2021_355_MOESM1_ESM.docx]

| **Table 1.** **Literature search strategy.** | | |
| --- | --- | --- |
| **Database** | **Time span** | **Search strategy** |
| **Pubmed** | **from January 1, 2000 to July 1, 2020** | **((((((“Colorectal Neoplasms”[Mesh]) OR ((colorect* OR rect* OR colon*) AND (neoplas* OR carcinoma* OR tumor* OR tumour* OR cancer* OR malignan* OR oncol*))))) AND (((“Stents”[Mesh] OR stent*)) OR (prosthesis OR endoprosthesis OR SEMS OR “self-expanding metal” OR tube OR “transanal drainage tube” OR “decompression tube” OR “ileus tube” OR colostomy OR colostomies OR “elective surgery”))) AND (bridge to surgery)))** |
| **Embase (Ovid), Medline (Ovid), Web of Science and Cochrane Library databases are searched using the similar search strategies mentioned above.** | | |

| **Table 2. Assessment of Methodological Quality of Studies Included** | | | | | | | | |
| --- | --- | --- | --- | --- | --- | --- | --- | --- |
| Trial | Study design | Random sequence generation | Allocation concealment | Assessment of blinding | Incomplete outcome data | Selective reporting | Other bias | NOS score |
| Arezzo, Alberto 2017 | RCT | Low risk | Low risk | Unclear risk | Low risk | Low risk | Low risk | - |
| Ghazal, A. H. 2013 | RCT | Low risk | Low risk | Unclear risk | Low risk | Low risk | Low risk | - |
| Ho, K. S 2012 | RCT | Low risk | Unclear risk | Unclear risk | Low risk | Low risk | Low risk | - |
| Pirlet, Isabelle A 2011 | RCT | Low risk | Low risk | Unclear risk | Low risk | Low risk | Low risk | - |
| Sloothaak, D. A 2014 | RCT | Low risk | Unclear risk | Unclear risk | Low risk | Low risk | Low risk | - |
| Jeanin E van Hooft 2011 | RCT | Low risk | Low risk | Unclear risk | Low risk | Low risk | Low risk | - |
| Cheung, H. Y 2009/2012 | RCT | Low risk | Low risk | Unclear risk | Low risk | Low risk | Low risk | - |
| Okuda, Y 2019 | Non-RCT | - | - | - | - | - | - | 8 |
| J. K. Jiang 2008 | Non-RCT | - | - | - | - | - | - | 8 |
| Amelung, F. J 2016 | Non-RCT | - | - | - | - | - | - | 8 |
| Mege, D. 2019 | Non-RCT | - | - | - | - | - | - | 8 |
| Veld, J. V 2020 | Non-RCT | - | - | - | - | - | - | 7 |
| Kagami, S 2018 | Non-RCT | - | - | - | - | - | - | 7 |
| Ryuichiro Sato 2019 | Non-RCT | - | - | - | - | - | - | 7 |
| Yang, Leilei 2019 | Non-RCT | - | - | - | - | - | - | 7 |
| Masayoshi Hosono 2019 | Non-RCT | - | - | - | - | - | - | 7 |
| Kawachi, Jun 2018 | Non-RCT | - | - | - | - | - | - | 7 |
| Amelung, F. J 2016 | Non-RCT | - | - | - | - | - | - | 7 |
| Oistamo, Emma 2016 | Non-RCT | - | - | - | - | - | - | 7 |
| Tanis, Pieter J 2015 | Non-RCT | - | - | - | - | - | - | 7 |
| Amelung, F. J 2017 | Non-RCT | - | - | - | - | - | - | 8 |
| Chen, X. Q 2019 | Non-RCT | - | - | - | - | - | - | 7 |
| Choi, J. M 2014 | Non-RCT | - | - | - | - | - | - | 8 |
| Consolo, P 2017 | Non-RCT | - | - | - | - | - | - | 7 |
| Erichsen, R 2015 | Non-RCT | - | - | - | - | - | - | 7 |
| Flor-Lorente, B 2017 | Non-RCT | - | - | - | - | - | - | 8 |
| Gorissen, K. J.2013 | Non-RCT | - | - | - | - | - | - | 7 |
| Han, Lijiang 2020 | Non-RCT | - | - | - | - | - | - | 7 |
| Ho, K. M. 2017 | Non-RCT | - | - | - | - | - | - | 9 |
| Kavanagh, D. O. 2013 | Non-RCT | - | - | - | - | - | - | 8 |
| Kim, M. K 2016 | Non-RCT | - | - | - | - | - | - | 8 |
| Kim, S. J. 2015 | Non-RCT | - | - | - | - | - | - | 7 |
| Kwak, M. S. 2016 | Non-RCT | - | - | - | - | - | - | 8 |
| Lee, G. J. 2013 | Non-RCT | - | - | - | - | - | - | 8 |
| Lim, T. Z. 2017 | Non-RCT | - | - | - | - | - | - | 8 |
| Lovero, R. 2020 | Non-RCT | - | - | - | - | - | - | 8 |
| Morita, S 2019 | Non-RCT | - | - | - | - | - | - | 8 |
| Park, J. 2018 | Non-RCT | - | - | - | - | - | - | 8 |
| Park, Sun Jin 2016 | Non-RCT | - | - | - | - | - | - | 7 |
| Rodrigues-Pinto, E 2019 | Non-RCT | - | - | - | - | - | - | 7 |
| Sabbagh, C 2013 | Non-RCT | - | - | - | - | - | - | 7 |
| van den Berg, M. W 2014 | Non-RCT | - | - | - | - | - | - | 7 |
| Yan, F. H. 2017 | Non-RCT | - | - | - | - | - | - | 7 |
| Yang, S. Y 2019 | Non-RCT | - | - | - | - | - | - | 7 |
| van den Berg, M. W 2014 | Non-RCT | - | - | - | - | - | - | 8 |
| Yan, F. H. 2017 | Non-RCT | - | - | - | - | - | - | 7 |
| Yang, S. Y 2019 | Non-RCT | - | - | - | - | - | - | 7 |
